# Profiles of objective and subjective cognitive function in Post-COVID Syndrome, COVID-19 recovered, and COVID-19 naïve individuals

**DOI:** 10.1038/s41598-024-62050-x

**Published:** 2024-06-11

**Authors:** A. R. Bland, M. Barraclough, W. R. Trender, M. A. Mehta, P. J. Hellyer, A. Hampshire, I. K. Penner, R. Elliott, S. Harenwall

**Affiliations:** 1 Department of Psychology, Manchester Metropolitan University, Manchester, UK; 2 Division of Musculoskeletal and Dermatological Sciences, School of Biological Sciences, Faculty of Biology, Medicine and Health, The University of Manchester, Manchester, UK; 3 NIHR Manchester Biomedical Research Centre, Manchester Academic Health Science Centre, Manchester University NHS Foundation Trust, Manchester, UK; 4 Department of Brain Sciences, Imperial College London, London, UK; 5 Institute of Psychiatry, Psychology and Neuroscience, King’s College London, London, UK; 6 Department of Neurology, Inselspital, Bern University Hospital, University of Bern, Bern, Switzerland; 7 Division of Psychology and Mental Health, School of Health Sciences, University of Manchester, Manchester, UK; 8 Primary Care Wellbeing Service, Bradford District Care NHS Foundation Trust, Bradford, UK

**Keywords:** Objective cognition, Subjective cognition, Post-COVID, Long-COVID, Fatigue, Stress, Cognitive control, Human behaviour

## Abstract

Post-COVID Syndrome has emerged as a significant public health concern worldwide with increasing evidence to suggest that individuals who have had an acute COVID-19 infection report lingering memory and attention difficulties, even in individuals who have fully recovered and no longer experiencing symptoms of COVID-19. The present study sought to investigate the profile of objective and subjective cognitive difficulties in people who have Post-COVID Syndrome, people who have fully recovered from an acute COVID infection and people who have never had COVID-19. We further sought to explore the extent to which self-reported fatigue and stress are related to subjective and objective cognitive difficulties. 162 participants including 50 people living with Post-COVID Syndrome, 59 people who have had COVID-19 but have fully recovered and 53 people who have never experienced symptoms of COVID-19 and had never tested positive for COVID-19 were recruited from Academic Prolific to complete a series of online questionnaires and neurocognitive tasks. Subjective cognitive function was measured using the Cognitive Failures Questionnaire and objective cognitive function was measured using the Cognitron cognitive test battery. We found that objective and subjective measures of cognitive function were not significantly related, suggesting that self-reports of “brain fog” are not reflecting objectively measured cognitive dysfunction. A MANOVA revealed that subjective cognitive deficits were driven by heightened perceived stress and fatigue and not significantly related to COVID-19 status. Objective cognitive function, however, was significantly related to perceived stress and COVID status whereby we observed significant objective cognitive deficits in people who have been exposed to an acute COVID-19 infection regardless of whether they had Post-COVID Syndrome or had fully recovered, as compared to people who had never had COVID-19. This suggests that an acute infection can have long term effects on cognitive function, even without persistent COVID-19 symptoms. Encouragingly, objective cognitive function was significantly associated with time since initial infection showing that cognitive deficits improved over time for people who had recovered from COVID-19. However, we did not observe the same improvement in individuals with Post-COVID Syndrome and observed that cognitive dysfunction was significantly related to the number of neurological symptoms presently experienced. These results add to the accumulating literature that COVID-19 is associated with significant cognitive difficulties following a COVID-19 infection, which appear to improve over time for those who have recovered from COVID-19 yet persist in people living with Post-COVID Syndrome.

## Introduction

Post-COVID Syndrome, a term used to describe the persistent symptoms experienced by individuals recovering from severe acute respiratory syndrome coronavirus 2 (SARS-CoV-2), has emerged as a significant public health concern worldwide. Prevalence estimates suggest that 45% of COVID-19 survivors, regardless of hospitalisation status, are experiencing a range of unresolved symptoms at ∼ 4 months^1^ and there is compelling evidence of an increased risk of long-term neurologic disorders^2^. Particularly, it has been reported that many individuals who have recovered from an acute COVID-19 infection experience “brain fog”, including problems with headaches, concentrating, disorientation and difficulty finding the right words^2–4^ with recent meta-analyses estimating that lingering memory and attention difficulties are evident in 22–32% of cases^5,6^.

Strikingly, even people who have fully recovered from COVID-19, including those no longer reporting symptoms, have been shown to exhibit significant cognitive deficits measured using objective cognitive tasks, versus people who had not been exposed to COVID-19^7^. This is in line with accumulating evidence that COVID-19 is associated with neural damage^8,9^, with markers of brain injury having been found in hospitalised COVID-19 patients^10^. Indeed, a UK Biobank study comparing magnetic resonance images (MRI) and objective cognitive tests recorded before and after SARS-CoV-2 infection found structural brain changes and longitudinal decline in cognitive performance^11^. Also, a large longitudinal neuroimaging study of individuals with mild to moderate COVID-19 showed a significant COVID-related reduction of grey matter thickness and volume in the left parahippocampal gyrus, orbitofrontal cortex and insula extending to the anterior cingulate cortex and temporal pole^11^—brain areas directly linked to attention, memory and executive processes. However, at present there is little evidence of how these objective cognitive dysfunctions are related to subjective self-reports of cognitive difficulties following COVID-19.

Subjective and objective cognitive function are two distinct measures of cognition. Subjective cognitive function refers to an individual's personal perception, awareness, and evaluation of their cognitive abilities, involving self-assessment of memory, attention, language, problem-solving, and decision-making^12^. Alternatively, objective cognitive function involves the measurement and evaluation of cognitive abilities using standardised neurocognitive tasks and clinical assessments. Research has shown that subjective and objective cognitive function are only moderately correlated but not interchangeable, with several studies having demonstrated no correlation between these measures in healthy individuals^13^ or in various patient groups known to experience cognitive difficulties, including systemic lupus erythematosus^14,15^, multiple sclerosis^16–18^ and Myalgic Encephalomyelitis (ME)/chronic fatigue syndrome (CFS)^19^. Typically, people evaluate their cognitive functioning as being worse than their objective performance on neuropsychological assessments, with some studies failing to find any objective cognitive impairments in groups of patients with post-viral chronic fatigue following Epstein-Barr virus^20^ and Q-fever^21^. Importantly, whilst some studies have demonstrated that objective cognitive impairment is mainly related to patients’ demographic and clinical factors, subjective cognitive impairment is often confounded by emotional state such as stress, anxiety and depression^16,22,23^. Indeed, a meta-analysis observed consistent associations between subjective cognitive function and affective symptoms^24^. However, the relationship between- and underlying drivers of- objective and subjective dysfunction in individuals recovering from COVID-19 is largely unknown. A recent study of Intensive Care Unit (ICU) COVID-19 survivors found that subjective cognitive impairments were found to be related to anxiety, depression and post-traumatic stress whereas objective measures were related to age and cognitive reserve, with little correspondence between the two^25^. However, this study did not utilise a control group or separate individuals who had Post-COVID syndrome versus those who have fully recovered from their acute infection. Given that subjective and objective cognitive function may be driven by different underlying mechanisms, it is important to explore factors which contribute to objective cognitive impairments and subjective feelings of “brain fog” in order to design targeted interventions for individuals living with Post-COVID. Factors which have previously been shown to contribute to the accuracy of subjective cognitive functioning include fatigue^26^ and affective symptoms^24^, aspects which are known to be exacerbated in individuals living with Post-COVID Syndrome^27^. Stress, in particular, is a symptom which is highly prevalent in Post-COVID individuals^28^ but is also recognised as heightened across the general population in response to the COVID-19 pandemic^29^. Indeed, daily stressors can produce transient effects on cognition by reducing the amount of attentional resources available for information processing^30,31^ and prolonged or excessive cortisol release can have deleterious effects on regions involved in cognitive processes, such as the prefrontal cortex and hippocampus^32^.

Taken together, understanding the relationship between subjective and objective cognitive impairment and the driving factors underlying these is crucial for the development of tailored rehabilitation programs aimed at improving cognitive function and facilitating recovery in Post-COVID patients. In line with previous literature in other patient groups, we hypothesised that subjective cognitive dysfunction would be associated with increased fatigue and stress whereas objective cognitive function would be most dominantly linked to clinical features of COVID-19.

## Results

Participants within the three groups were well-matched with regards to age, sex and years in education (Table 1). The majority of Post-COVID syndrome participants (Post-COVID) and participants who had recovered from an acute COVID-19 infection (Recovered-COVID) had their acute COVID-19 infection confirmed by a positive PCR or lateral flow test (90%) with the remainder reporting that tests were not available at the time of acute infection. On average, participants were one year from their first diagnosis with some participants experiencing an acute infection 2 or even 3 times. Overall, results demonstrated that individuals who had an acute COVID-19 infection regardless of whether they had Post-COVID syndrome or had fully recovered (COVID +), showed significantly greater fatigue, stress, subjective cognitive difficulties, and objective cognitive difficulties, specifically in the Tower of London, Spatial Span and Object Memory compared to those who had not had COVID-19 (COVID-) (see Table 1). In line with previous literature, objective cognitive function did not significantly correlate with objective cognitive function (*r* = − 0.07, *p* = 0.161).

**Table 1:** descriptive statistics for each variable.

Measure		Means			ANOVA	t-test
		Post COVID	Recovered COVID	No COVID	PC v RC v NC	COVID + v COVID-
Demographic	Age	41.66	38.12	41.98	Ns	Ns
	Sex (female)	58%	62.1%	47.2%	Ns	Na
	Years in Education	15.56	16.19	15.53	Ns	Ns
COVID-19	Time since first diagnosis (months)	10.48	12.83	na	Na	Ns
	Positive COVID-19 test	96%	89.7%	na	Na	Ns
	Hospital admission	18%	10%	na	Na	Ns
	COVID-19 more than once	18%	18.9%	na	Na	Ns
Covariates	Fatigue	65.24	50.31	50.36	PC > RC***, PC > NC***	COVID + > COVID-**
	Stress	18.36	15.84	15.62	PC > RC*,	ns
Subjective CF	Distractibility	15.04	12.14	11.70	PC > NC**, PC > RC*	COVID + > COVID-*
	Forgetfulness	16.78	12.37	11.74	PC > NC***, PC > RC ***	COVID + > COVID-**
	False triggering	12.16	8.90	8.23	PC > NC**, PC > RC ***	COVID + > COVID-**
	CogFail total	44.42	34.71	32.66	PC > NC**, PC > RC ***	COVID + > COVID-**
Objective CF	Block rearrange	9.55	9.24	9.77	ns	ns
	Tower of London	4.37	4.59	5.30	PC > NC = 0.055	COVID + > COVID-*
	Spatial Span	5.78	6.05	6.32	PC < NC*	COVID + > COVID-*
	Target Detection	55.87	55.93	57.43	Ns	Ns
	Object Memory—I	51.70	54.22	66.32	PC < NC***, RC < NC**	COVID + > COVID-***
	Object Memory—D	47.80	50.51	60.47	PC < NC**, RC < NC**	COVID + > COVID-***
	Global score	-0.17	-0.13	0.30	PC < NC**, RC < NC**	COVID + > COVID-**

PC = Post-COVID, RC = Recovered from COVID-19, NC = Never had COVID-19. COVID +  = have had an acute COVID-19 infection in the past (PC and RC), COVID- = have never had COVID-19. ***p < 0.001, **p < 0.01, *p < 0.05, *Ns* = non-significant; *Na* = not applicable.

### Objective and subjective cognitive function in Post-COVID, Recovered-COVID and No-COVID

**Figure 1:**
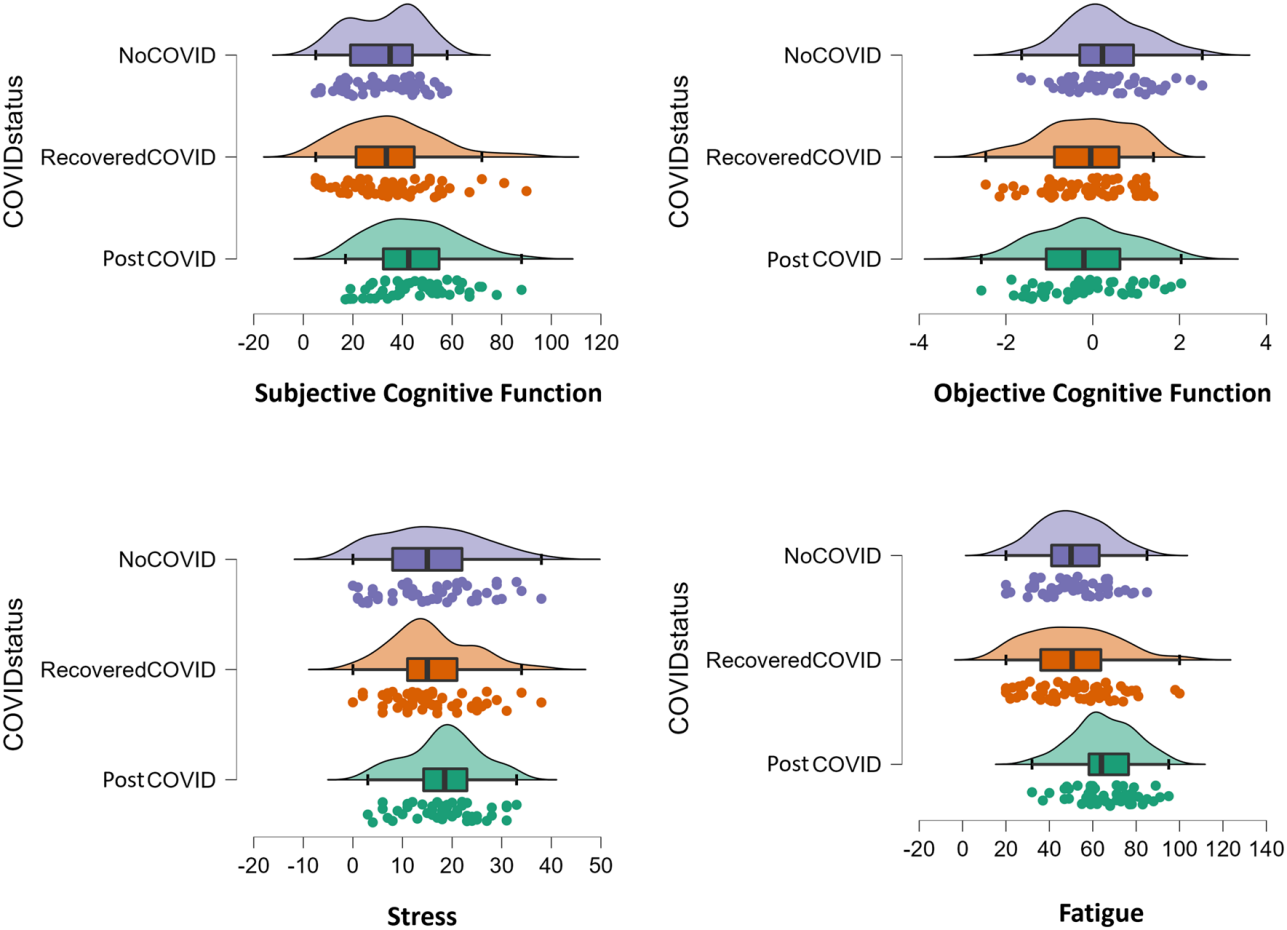
Raincloud plots for subjective and objective cognitive function across COVID groups. A raincloud data visualization approach^33^ which combines an illustration of data distribution (the ‘cloud’), with jittered raw data (the ‘rain’) and a boxplot to visualize central tendency and error. Subjective cognitive Function = Cognitive failures Questionnaire whereby a higher score indicated greater self-reported distractibility, forgetfulness and false triggering. Objective Cognitive Functioning = Global Index Score of 5 Cognitron Tasks whereby a greater score indicates greater cognitive performance.

A MANCOVA was conducted to determine whether there was a significant difference between the three COVID groups on subjective and objective measures of cognitive function. Overall, there was significant effect of COVID group (F(4, 312) = 2.70, *p* < 0.001, partial eta squared = 0.07). Subjective cognitive test scores revealed significant differences between the three groups (*F*(2,156) = 7.44, *p* < 0.001, *η*^2^ = 0.09) showing that Post-COVID participants reported greater subjective cognitive difficulties compared to Recovered-COVID (*p* = 0.006, CI = 2.29, 18.09) and No-COVID participants (*p* < 0.001 , CI = 3.75, 19.72). There was no significant difference in subjective cognitive function between Recovered-COVID and No-COVID participants (*p* = 1.000, CI = -9.34, 6.25). Alternatively, objective cognitive tests scores revealed a significant main effect of group (*F*(2,156) = 4.47, *p* = 0.013, *η*^2^ = 0.05) showing that that both Post-COVID (*p* = 0.038, CI = 0.02, 0.94) and Recovered-COVID (*p* = 0.026, CI = 0.04, 0.95) participants demonstrated poorer performance compared to No-COVID participants (see Fig. 1).

### Fatigue, stress, and subjective cognitive function

Entering fatigue and stress as covariates into the MANCOVA revealed an overall significant main effect of stress (F(2, 153) = 4.70, *p* = 0.011, partial eta squared = 0.06) and a significant effect of fatigue (F(2, 154) = 36.61 p < 0.001,partial eta squared = 0.32). The significant main effect of COVID group on subjective cognitive performance was no longer significant when controlling for fatigue and stress (*F*(2,154) = 0.56, *p* = 0.575, *η*^2^ = 0.01) suggesting that greater self-reported cognitive dysfunction in Post-COVID-19 compared to Recovered-COVID and No-COVID participants is not related to COVID-19 status when taking into account heightened fatigue (*F*(2,154) = 72.85, *p* < 0.001, *η*^2^ = 0.32) and stress (*F*(2,154) = 4.62, *p* = 0.033, *η*^2^ = 0.03).

### Fatigue, stress, and objective cognitive function

Stress showed a significant effect on objective cognition (*F*(2,154) = 4.75, *p* = 0.031, *η*^2^ = 0.03), but fatigue did not (*F*(2,154) = 0.98, *p* = 0.325, *η*^2^ = 0.01). Nevertheless, the main effect of COVID group on objective cognition remained significant (*F*(2,154) = 4.61, *p* = 0.011, *η*^2^ = 0.06) whereby both Post-COVID (*p* = 0.04 , CI = 0.08, 0.98) and recovered-COVID participants (*p* = 0.023, CI = 0.05, 0.95) demonstrated significantly poorer performance compared to No-COVID participants.

### Effects of clinical variables

**Figure 2:**
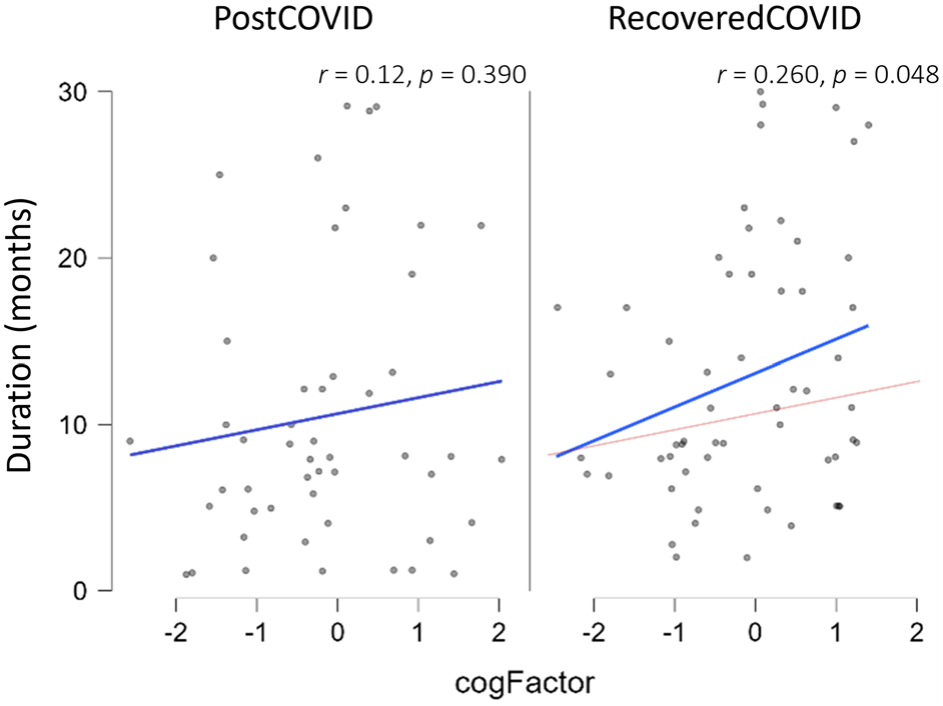
Scatter plots showing the relationship between duration (months) since initial COVID-19 infection and objective cognitive function in people with Post-COVID and people who have recovered from COVID. Objective Cognitive Functioning = Global Index Score of 5 Cognitron Tasks (cogFactor) whereby a greater score indicates greater cognitive performance. Red line illustrates a ghost line of line of best fit for the Long-COVID group.

Entering COVID-19 confirmation with a PCR as a covariate did not impact either subjective (*p* = 0.747) or objective cognitive functioning (*p* = 0.636). Similarly entering hospitalisation as a covariate did not impact either subjective (*p* = 0.787) or objective cognitive functioning (*p* = 0.240). Encouragingly, entering time from diagnosis into the model showed that objective cognitive functioning significantly improved with greater time from diagnosis (*F*(1,101) = 4.55, *p* = 0.035, *η*^2^ = 0.04). This however, appeared to be significant only for Recovered-COVID participants (*r* = 0.26, *p* = 0.048) and not Post-COVID participants (*r* = 0.12, *p* = 0.390). No such improvement was observed in subjective cognitive functioning (*F*(1,155) = 0.158, *p* = 0.692, *η*^2^ = 0.002) whereby participants perceptions of cognitive dysfunction were not improving in line with objective improvements. Our results show that whilst objective cognitive function improves over time in those who are no longer experiencing symptoms of COVID-19, individuals with persistent symptoms do not experience the same recovery over time (Fig. 2).

### Post-COVID symptoms

**Figure 3:**
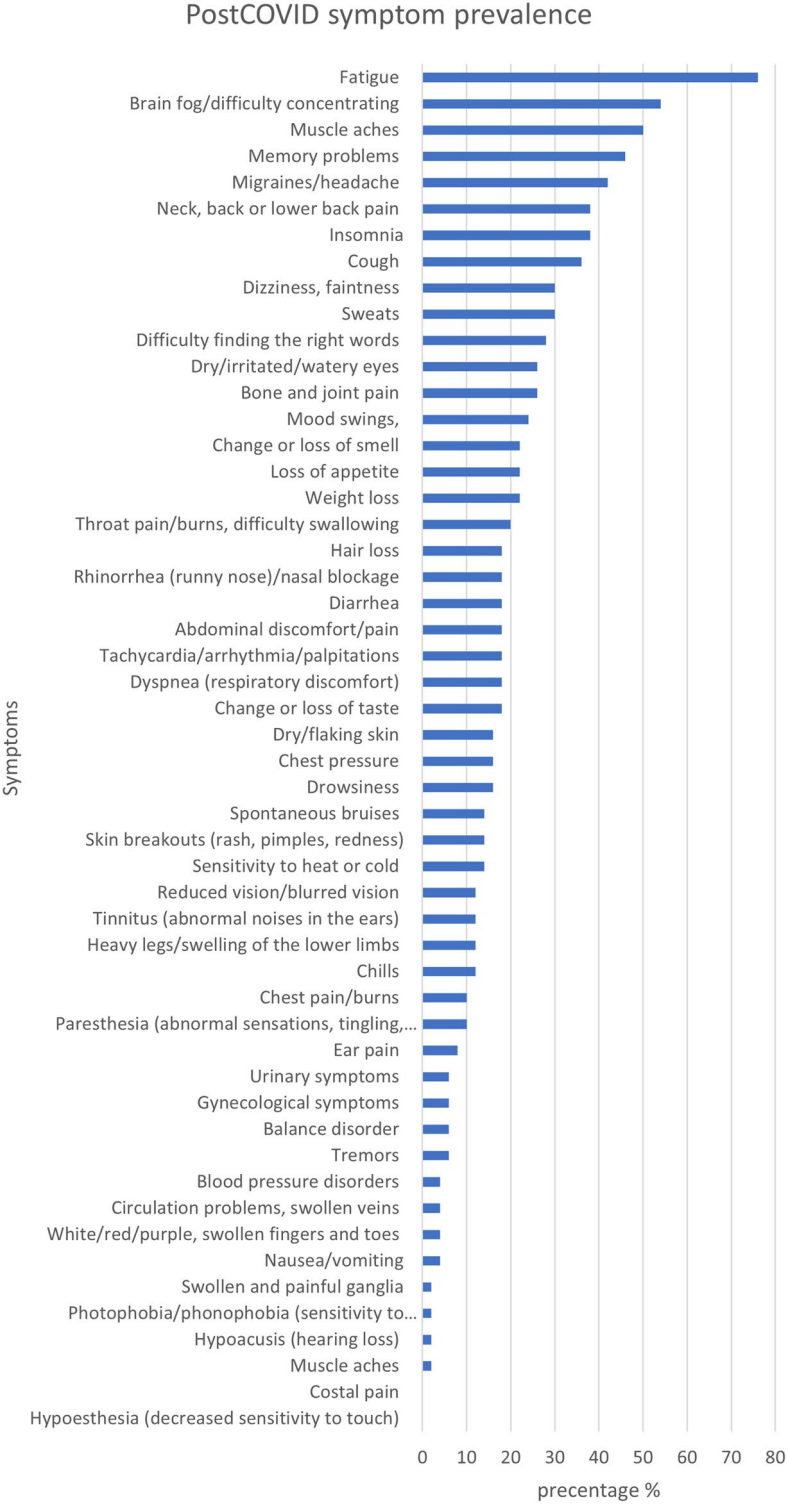
Percentage of participants self-reporting each symptom in the Post-COVID symptom tool^34^.

**Figure 4:**
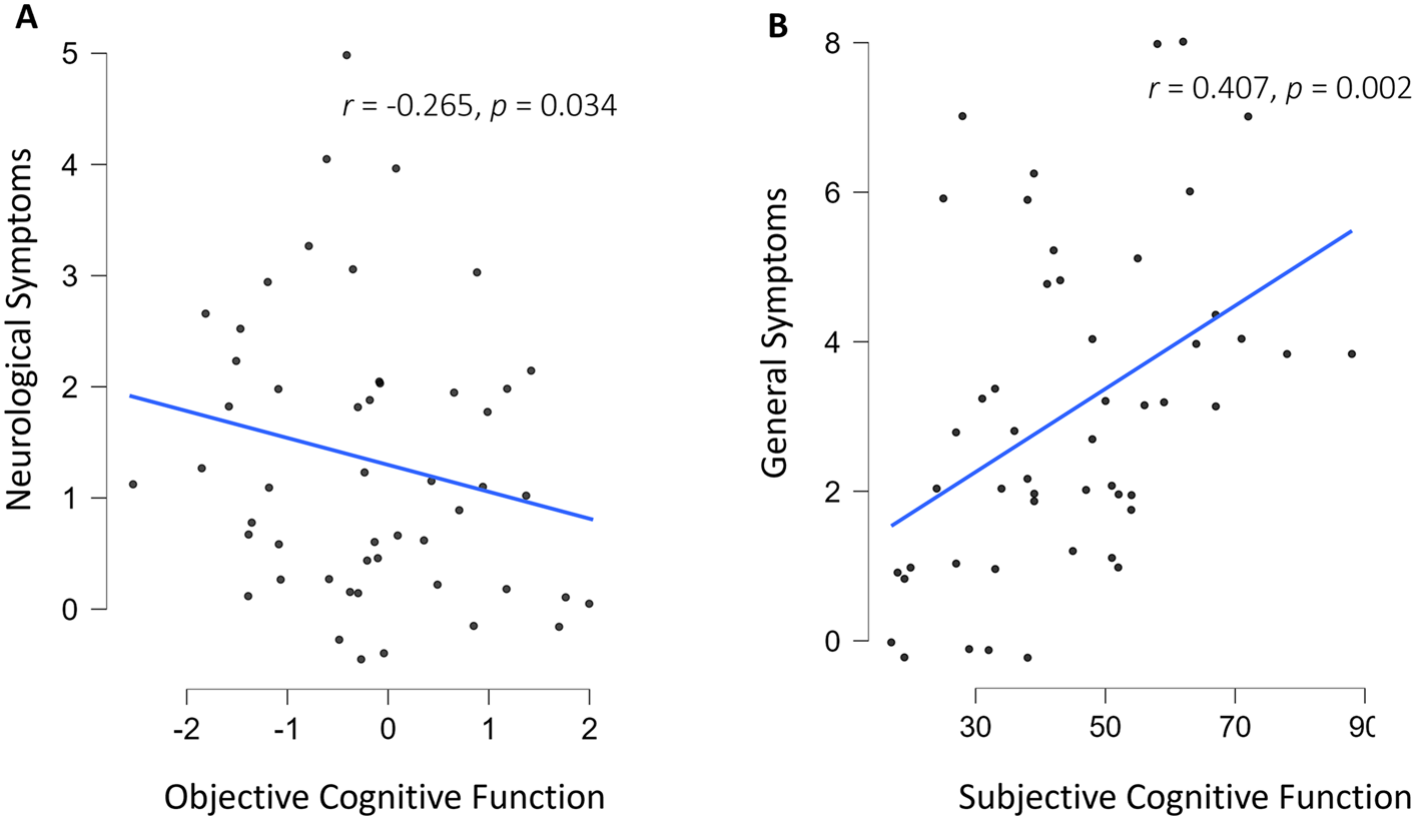
(**A**) Scatter plot showing the significant relationships between objective cognitive function and neurological symptoms. (**B**) Scatter plot showing the significant relationships between subjective cognitive function and general symptoms. Symptoms were measured using the Post-COVID symptoms and Impact Tools^34^. Subjective cognitive Function = Cognitive failures Questionnaire whereby a higher score indicated greater self-reported distractibility, forgetfulness and false triggering. Objective Cognitive Functioning = Global Index Score of 5 Cognitron Tasks whereby a greater score indicates greater cognitive performance.

We utilised the Post-COVID symptom tool^34^ to assess the number of symptoms experienced by the Post-COVID group (Fig. 3) and to explore the relationship between subjective and objective cognitive function and Post-COVID symptomatology (Fig. 4).

In order to explore neurological symptoms we excluded three items which related to brain fog in order to prevent confounding with subjective cognitive dysfunction. These included problems with *brain fog/difficulty concentrating*, *memory problems* and *difficulty and finding the right words*. The remaining neurological items included; *migraines/headache, dizziness, faintness, paresthesia (abnormal sensations, tingling, burning, *etc*.), change or loss of smell, change or loss of taste, hypoesthesia (decreased sensitivity to touch), tremors* and *balance disorder.*

Whilst controlling for age and years of education, we found that objective cognitive function was significantly correlated with the number of neurological symptoms experienced in the last 30 days (Fig. 4: *r* = − 0.265, *p* = 0.034). Objective cognitive function, however, was not significantly associated with any other group of symptoms assessed by the tool, including: general (*r* = − 0.033, *p* = 0.412), thorax (*r* = − 0.033, *p* = 0.413), musculoskeletal (*r* = 0.135, *p* = 0.820), digestive (*r* = 0.084, *p* = 0.714), ear nose and throat (*r* = − 0.137, *p* = 0.176), skin and hair (*r* = − 0.194, *p* = 0.093), eyes, vessels and ganglia (*r* = 0.162, *p* = 0.865) or urinary and genital (*r* =− 0.054, *p* = 0.358). Alternatively, subjective cognitive function was significantly associated with general Post-COVID symptoms (*r* = 0.407, *p* = 0.002) which included fatigue, chills, sleep problems, mood swings, muscle aches, weight loss, loss of appetite, sweats, drowsiness, sensitivity to heat or cold and hot flashes.

## Discussion

The present study found significant cognitive deficits, specifically in spatial problem solving, working memory and object episodic memory, as measured by objective neurocognitive tasks in people who have been exposed to an acute COVID-19 infection regardless of whether they were living with Post-COVID or had fully recovered. The present study also found that objective and subjective measures of cognitive function were not significantly correlated, and that self-reported experience of cognitive deficits were no longer significant when accounting for heightened stress and fatigue. Importantly, however, objective cognitive deficits, although related to stress, could not be explained by stress or fatigue but were significantly related to COVID19 status. This suggests that differences in objective cognitive function are significantly related to a history of an acute COVID infection. Finally, objective cognitive impairment appears to improve over time for people who have recovered from COVID, whereas individuals with Post-COVID do not demonstrate the same improvements. We observed a significant correlation between an increasing number of neurological symptoms and greater cognitive difficulties, suggesting that ongoing neurological symptoms in Post-COVID syndrome continue to impact cognitive function.

The findings of the present study are consistent with previous studies examining COVID-19 related cognitive dysfunction showing that cognitive dysfunction persists even after full recovery from an acute COVID-19 infection^7^. We found that the most significant cognitive dysfunction was specific to the object memory task which assesses visual episodic memory. This is consistent with previous studies utilising these cognitive tasks which have shown no differences in working memory, executive function, planning and mental rotation in people fully recovered from COVID-19, yet they show significantly worse episodic memory^35^. In addition to this, our Post-COVID group showed significantly poorer performance in spatial span, a test designed to measure spatial short-term memory capacity and a trend towards poorer performance in the Tower of London designed to test measures of spatial planning and executive function. These findings highlight both the initial infection and subsequent persistent symptoms have an impact on cognitive function.

In line with previous studies, we also found evidence to suggest that cognitive dysfunction was improving over time for people who had fully recovered from COVID-19. Indeed, Zhao et al.^35^found that cognitive function was not significantly different from normative scores after 6–9 months, demonstrating evidence of recovery over time. Indeed, recent large scale data does suggest that COVID-19 related neurological symptoms do generally decline over time^36^. However, in the present study we show that cognitive improvement is not significant for those living with Post-COVID Syndrome, suggesting that persistent symptoms of the virus interfere with full cognitive recovery. Specifically, the average time from first COVID-19 infection was 11 months with 18% of participants reporting repeat infections suggesting that full recovery time may potentially take years. Likewise Rass et al. ^37^ and Taruffi et al.^38^, shows that symptoms tend to persist at 3-month and 1-year follow-ups, with some longitudinal data showing that deficits persisted at almost two years since infection^39^.

We also found a significant effect of stress on objective cognitive dysfunction, albeit to a lesser degree when compared to subjective cognitive dysfunction. COVID-19 has introduced multiple stressors, including the fear of infection itself, social isolation, economic uncertainty, and grief from the loss of loved ones. These stressors have led to elevated levels of psychological distress among individuals globally^40^. Stress hormones such as cortisol, can have detrimental effects on the brain^41^. In particular, the physiological systems classically activated in response to stress, including the sympathetic nervous system, the hypothalamus–pituitary–adrenocortical (HPA) axis and central neurotransmitter and neuropeptide systems, impact brain regions that play central roles in cognitive function^42^. Furthermore, stress-related changes in neurochemical systems, such as the noradrenergic and dopaminergic systems can influence attention, arousal, and cognitive flexibility^43^, additionally contributing to cognitive impairments. Importantly, the relationship between cognitive dysfunction and neuroinflammation is bidirectional whereby neuroinflammation may contribute to cognitive deficits by inducing neuronal injury and synaptic dysfunction, conversely, stress-related cognitive impairments, can exacerbate neuroinflammation through activation of the hypothalamic–pituitary–adrenal (HPA) axis and release of stress hormones^44^.

Understanding the underlying mechanisms driving cognitive dysfunction is essential in order to design effective interventions for people living with persistent symptoms of COVID-19. There are several hypothesized mechanisms for Post-COVID pathogenesis, including immune dysregulation, microbiota disruption, autoimmunity, clotting and endothelial abnormality, and dysfunctional neurological signalling^45^. One study found that the most severely cognitively affected patients demonstrated hypometabolism in the frontoparietal regions^46^, brain regions which are implicated in sustained attention and visual episodic memory^35^. Whilst there is no known cure for Post-COVID syndrome, potential therapeutics to target pathogenic mechanisms include anti-inflammatory, anti-viral, and neuro-regenerative agents to reverse neurological sequelae^47^. It will also be important for future studies to understand any links between quality of life and subjective vs objective cognitive deficits to design tailored interventions. NICE recommends a multidisciplinary approach, with emphasis on lifestyle management approaches to optimise physical and psychological functioning alongside any appropriate medical interventions^27^. Indeed many lifestyle interventions have a growing evidence base for the direct impact on cognitive functioning including exercise^48^, nutrition^49^, mindfulness^50^ and other psychotherapy approaches, such as such as Eye Movement Desensitization and Reprocessing (EMDR) demonstrating potential for structural changes to the brain^51,52^. Taken together, understanding the overlap and divergence of objective and subjective cognitive function in people with Post-COVID Syndrome will enable the development for effective treatments and interventions.

### Limitations

This study has a number of limitations. Our sample size of 161 participants with 50–58 per group may not have been sufficient to detect more subtle effects. In particular, 18% of Post-COVID and 18% Recovered-COVID participants had more than one instance of an acute COVID infection therefore larger sample sizes would have allowed further investigation into whether multiple infections significantly impact subjective and objective cognitive difficulties. It is also important to note that this study relied on self-reported COVID-19 status and not medical records. In addition, participants who had a depression or anxiety diagnosis were excluded from this study in order to control for potential confounding effects on the measures of cognition. However, some individuals living with Post-COVID may have a clinical mental health diagnosis as a consequence of COVID-19 therefore this may lead to a bias in the sample that does not represent the wider population of people who have recovered and are recovering from COVID-19.

## Conclusion

Whilst it does appear that people who have recovered from COVID-19 show improvement over time in objective cognitive dysfunction, further investigation is needed into the underlying neural mechanisms in order to design effective treatments for individuals living with the persistent symptoms of Post-COVID. In conclusion, the emerging evidence of cognitive dysfunction in COVID-19 patients suggests long-term consequences of the virus that could have profound implications for affected individuals' quality of life. As such, future research should focus on longitudinal studies to assess the persistence and progression of cognitive dysfunction in COVID-19 survivors over time. Such investigations will help determine the extent of long-term cognitive impairment and inform strategies for rehabilitation and support.

## Methods

### Participants

Participants were excluded from the study if they had any previous or current clinically diagnosed psychiatric or neurological disorder. Twenty-two participants were excluded due to taking mood altering medication at the time of the study. Post-COVID participants were eligible for inclusion if they had experienced new or ongoing symptoms 12 weeks or more after an acute COVID-19 infection (NICE, 2020). One participant was excluded from the Post-COVID Syndrome group due to being less than 12 weeks from acute COVID-19. The final sample consisted of 162 participants including 50 Post-COVID Syndrome participants (Post-COVID; mean age: 41.66, 58% female), 59 people who have had COVID-19 but have fully recovered (Recovered-COVID; mean age: 38.12, 62% female) and 53 people who have never experienced symptoms of COVID-19 and had never tested positive for COVID-19 (No-COVID; mean age: 41.98, 47% female).

## Procedure

Participants were recruited online via Prolific (https://www.prolific.co/) and were reimbursed at a rate of £10 per hour. Participants were directed to the study using a QR code to access Qualtrics (www.qualtrics.com) to complete the questionnaires and subsequently redirected to the Cognitron tasks^7^. Participants completed the study remotely on either a phone, tablet or computer and were allowed an unlimited amount of time. This study was approved by the Manchester Metropolitan University (EthOS ID: 40,456) and all research was performed in accordance with relevant guidelines/regulations. All participants were provided with an information sheet and were required to give full informed consent before proceeding to the study questionnaires.

## Materials

### Questionnaires

#### Post-COVID symptoms and impact

All participants were initially screened using Academic Prolific to gather information regarding whether they have ever had any COVID-19 symptoms, have ever tested positive for COVID-19, how many times they have had COVID-19 and whether they are still experiencing ongoing symptoms after 12 weeks since the initial infection in order to assign to the appropriate groups. Post-COVID participants were asked about their symptoms and their impact using the Post-COVID symptom and impact tools which is a validated scale developed from PCS patients’ lived experience^34^. This tool has two parts (1) *Symptom tools* which scores the number of symptoms patients experienced over the last 30 days and has a range from 0 (no symptoms) to 53. The *Impact Tool* scores impact of PCS on their life, across personal activities, family, profession, social, mental wellbeing and caregivers, and has a range of 0 (no impact) to 60 (maximum impact). In this study, the measure demonstrated good internal consistency (α = 0.90).

## The fatigue scale for motor and cognitive functions

To measure fatigue, the Fatigue Scale for Motor and Cognitive Functions (FSMC)^53^ was administered to all participants. This questionnaire, originally designed to assess fatigue in Multiple Sclerosis (MS) has more recently been utilised to measure fatigue in Post-COVID^54^. The FSCM questionnaire has 20 items with a Likert-scale from 1 (does not apply at all) to 5 (applies completely). The FSMC generates three scores: One score for cognitive fatigue (score range from 10 to 50), another for motor fatigue (score range from 10 to 50), and a third for total fatigue (score range from 20 to 100) which sums up the two components motor and cognitive fatigue. Further the scale allows for a symptom grading into mild, moderate and severe. In this study, the measure demonstrated good internal consistency (α = 0.96).

## The perceived stress scale (PSS)

The most popular instrument to assess perceived stress is the Perceived Stress Scale (PSS)^55^. The 10-item version of the PSS measures the degree to which individuals appraise situations in their life as stressful in the last month^56^. Questions were answered on a Likert scale from 0 to 4 from ‘never’ to ‘very often’. The total scores were calculated so that higher total scores indicated greater stress; the sum score ranged from 0 to 40. In this study, the measure demonstrated good internal consistency (α = 0.93).

## Subjective and objective cognitive measures

### Subjective: cognitive failures questionnaire

Subjective cognitive function was evaluated using the Cognitive Failures Questionnaire (CFQ)^57^, a 25-item questionnaire about minor mistakes in daily life over the last 2 weeks. For example: ‘Do you find you forget appointments?’ ‘Do you fail to notice signposts on the road?’ ‘Do you find you forget which way to turn on a road you know well but rarely use?’. In this study, the measure demonstrated good internal consistency (α = 0.94).

## Objective: selected cognitron tasks

The cognitive tests included in this study are part of the Cognitron cognitive test battery^7^ and can be viewed at https://oxmh1.cognitron.co.uk. These tasks have been shown to be robust across devices, sensitive to population variables of interest such as age, sex and education level. The tasks we used were Block Rearrange, Tower of London, Object Memory, Spatial Span and Target Detection, as described below. These Cognitron tasks have been utilised in several studies investigating cognitive difficulties in COVID-19^7,35,39^.

## Block rearrange

The Block Rearrange test measures spatial problem solving. Participants were presented with a grid of coloured blocks on the left-hand side of the screen and on the right-hand side, a black silhouette made up of a subset of the shapes on the left. The participant must make the shape of the left-hand blocks match the silhouette on the right-hand side by removing blocks. The blocks fall under gravity. The test comprises 15 trials of varying difficulty. The difficulty is modulated by two factors; the number of blocks needed to be removed and the number of blocks that must fall in order to reach the target silhouette. Each trial is terminated if either the target silhouette is reached (correct trial) or an incorrect block is removed (incorrect trial). The outcome measure is the total number of correct trials.

## Tower of London

This task measured spatial problem solving. Participants were shown two sets of three prongs with coloured beads on them. The first set was the initial state and the second set was the target state. The task was to work out the lowest number of moves it would take to transition from the initial state to the target state, then input this number using an on-screen number pad. The test consisted of 10 trials of variable difficulty, scaled using the number of beads and the convolutedness, defined as the number of moves that must be made that do not place a bead in its final target position. The outcome measure was the total number of correct trials.

## Object episodic memory

This task measured short term memory capacity. Participants were asked to memorize 20 images of everyday objects, depicted in black and white. The images were presented sequentially in a random order, each for 2000ms with an inter-image interval of 500ms. Immediately after the presentation, participants’ memory of the 20 images was tested in 20 randomly ordered trials. Each trial required participants to select a previously displayed image from a set of eight images; incorrect images differed in the object itself, look or orientation in order to measure not only whether the correct target was identified, but also at higher precision the similarity of selected objects to the original target when errors were made. Participants memory was tested again at the end of the experiment, without being shown the target objects again.

## Spatial span

This task measured working memory capacity. Participants were presented with a 4 × 4 grid, where each individual cell can light up sequentially. Participants were required to memorize the sequence and replicate it by clicking the appropriate squares in the order they lit up. The difficulty was incremented using a ratchet system, every time a sequence was recalled correctly, the length of the subsequent sequence was incremented by one. The test was terminated when three consecutive mistakes were made on a particular sequence length. The outcome measure was the maximum sequence length correctly recalled (ranging from 2 to 16).

## Target detection

This task measured spatio-visual attention. Participants were presented with a target shape on the left of the screen and a probe area on the right side of the screen. After 3 s, the probe area began to fill with shapes, the participant must identify and click the target shape whilst ignoring the distractor shapes. Shapes were added every 1 s and a subset of the shapes in the probe area is removed every 1 s. The trial ran for a total of 120 addition/removal cycles. The target shape was included in the added shapes pseudo-randomly, at a frequency of 12 in 20 cycles. The outcome measure was the total number of target shapes clicked.

## Data analysis

Data was analysed using IBM SPSS Statistics Version 28 (IBM Corporation, 2021) and JASP (JASP Team (2020), version 0.14.1). The statistical significance level was set to *p* < 0.05 (one-tailed). In order to explore subjective cognitive functioning between the groups we calculated the total score for subjective cognition (CogFail questionnaire). For the objective measure, we applied principal component analysis (PCA) to the five test summary scores, to derive a global index, in line with previous use of Cognitiron tasks^39^. A Multivariate Analysis of Covariance (MANCOVA), was conducted with COVID group as the independent variable, and total subjective and objective cognitive functioning as the dependent variables. Age and years in education (derived from highest qualification) were entered as covariates in all analyses, in order to control for age and education related effects of cognitive performance. Subsequently, fatigue and stress measures were entered as further covariates in the model to establish whether differences in cognitive function were impacted by fatigue and stress. Pillai's trace statistic was used, and all post-hoc tests were corrected using Bonferroni to control for multiple comparisons. There was no missing data for questionnaire measures, however two participants failed to complete the cognitive tasks in full. This data was estimated using the series mean before being entered into the PCA. No multivariate outliers were identified or removed from the data.

## Data Availability

Data is available on request from Dr Amy Bland (a.bland@mmu.ac.uk).
